# Relationship Between Intelligence and Creative Potential: Evidence from Data-Driven Analysis

**DOI:** 10.3390/jintelligence14060097

**Published:** 2026-06-02

**Authors:** T. W. Chiang, Angela F. Y. Siu, Alexis T. M. Pang, Samson S. S. Chan, Jeff T. K. Ng, Pamelia L. F. Ng

**Affiliations:** Program for the Gifted and Talented, Faculty of Education, The Chinese University of Hong Kong, ShaTin, New Territories, Hong Kong SAR, China; tinwaichiang@cuhk.edu.hk (T.W.C.); atmpang@cuhk.edu.hk (A.T.M.P.); samson@cuhk.edu.hk (S.S.S.C.); tszkitjeffng@cuhk.edu.hk (J.T.K.N.); lingfungpameliang@cuhk.edu.hk (P.L.F.N.)

**Keywords:** intelligence, creativity, divergent thinking, the threshold hypothesis

## Abstract

The relationship between intelligence and creativity is unclear despite the investigations that have been done over the decades. **(1) Background:** One of the most prominent beliefs on this relationship is the threshold hypothesis. This hypothesis assumes that at least above-average intelligence is necessary for high creativity, and the threshold is at the intelligence quotient (IQ) of 120. However, empirical research results on this hypothesis are limited and inconsistent. While earlier research supported that the hypothesis was subjected to doubts in methodology, recent studies that employed segmented regression analysis produced different breakpoints. **(2) Methods:** This study investigated the relationship between intelligence and one key component of creative potential (i.e., fluency) in 9358 Chinese students (53% male) aged six to twenty across twelve cohorts of an education program in five years through segmented regression analysis, employing the Davis test for unknown changepoints combined with bootstrap validation. **(3) Results:** After controlling confounding variables (i.e., cohort, gender, grade and age), the hypothetical breakpoint IQ 120 yielded non-significant results, failing to improve upon the positive linear baseline correlation between intelligence and creative potential. **(4) Conclusions:** Insights on a possible different threshold and three-breakpoint model were gained by using a data-driven approach.

## 1. Introduction

The relationship between intelligence and creativity is likely to be one of the most enduring yet contentious puzzles in the fundamental conception of human cognitive development. Guilford and Christensen (1973) hypothesized that intelligence is “an indicator of an upper limit for at least some creativity performance.” However, by using the scatter plot method, they found a continuous gradual shift from low to high IQ with no evidence to support any breaks. Therefore, they concluded that the higher the IQ, the more likely individuals are to have high creative potential. However, this is not the end of this threshold hypothesis debate. Researchers are still interested in seeking the potential threshold because it matters beyond academic debate if it exists. In the educational settings, if the intelligence–creativity relationship is a simple positive linear, then gifted programs could focus primarily on high-IQ identification. If intelligence and creativity are largely independent, then separate talent identification processes should be considered. Ambiguously enough, mixed results on this hypothesis were found since the introduction of this hypothesis. The current state of confusion could mean educational inequity and loss of human creative potential.

Ideally, we would possess clear empirical evidence for predicting creativity from intelligence. Reality falls short of this ideal. The more empirical studies we have conducted, the more diverse evidence we have gathered on this threshold hypothesis. Some researchers found no evidence of any thresholds (e.g., Guilford & Christensen, 1973; Sligh et al., 2005; Preckel et al., 2006; Holling & Kuhn, 2008; Sorjonen et al., 2019; Breit et al., 2023) while other researchers confirmed at least one threshold (e.g., Fuchs-Beauchamp et al., 1993; Cho et al., 2010; Neubauer et al., 2014; Karwowski & Gralewski, 2013; Shi et al., 2017; Çetinkaya, 2023). This puzzle becomes more confusing when we examine these intelligence–creativity relationships in different cultural contexts. It is possible that cultures approach creativity differently since Russian students, for instance, outperform their Emirati counterparts in creativity tests, even when their intelligence levels are lower (Repeykova et al., 2025). This pattern further suggests that intelligence may not drive creativity equally across cultures. Hence, whether the intelligence–creativity relationship changes when it meets the threshold, a phenomenon proposed by Western researchers, even if it exists, applies to all cultures remains doubtful.

### 1.1. Threshold Hypothesis

Guilford (1959) positioned divergent thinking (DT) squarely within his broader Structure of Intellect (SI) model, suggesting creativity and intelligence occupied overlapping rather than entirely disparate territories. Later, Sternberg and Lubart’s (1992) investment theory of creativity positioned intelligence, though explicitly not sufficient in itself, as one of several necessary ingredients for creativity. This “necessary but not sufficient” framing has become increasingly influential. Karwowski et al. (2016, 2017) found evidence to support this “necessary but not sufficient” condition through Necessary Condition Analysis (NCA, Dul, 2016), suggesting that intelligence might constitute a floor below which sustained creativity becomes unlikely, yet transcending that floor provides no guarantee of creative output.

#### 1.1.1. Intelligence–Creativity Relationship

Despite that there is evidence on the “necessary but not sufficient” condition, findings on intelligent-creativity relationships are still scattered. A landmark meta-analysis by Kim (2005) found an average correlation between intelligence and creativity of merely *r* = 0.17. This underwhelming association appeared to validate that intelligence and creativity, while related, diverge in their cognitive architecture. As methodological scrutiny intensified, more recent research has unveiled a slightly different landscape. A comprehensive meta-analysis by Gerwig et al. (2021) analyzing 849 correlations across 112 studies (*N* = 34,610) detected a corrected correlation of *r* = 0.25 between intelligence and creativity. A modest but consistently positive effect that climbed to *r* = 0.37 under specific conditions such as test-like settings, explicit “be-creative” instructions, and originality scoring methods. These results, while moderate, suggest intelligence plays a more pronounced role than Kim’s (2005) earlier estimate implied. Although these two large-scale meta-analyses found that intelligence and creativity are likely to be positively correlated, the modest correlation and great variations in the studies included still challenge the assumption of linearity on the intelligence–creativity relationship.

#### 1.1.2. Data Analytical Procedures

The assumption of linearity on the intelligence–creativity relationship influences data analytical choices. Weiss et al. (2020) criticized that the inconclusive results of previous hypothesis studies are possibly caused by using suboptimal data analytical procedures. Although *z* = 1.33 is always employed as a threshold, there is no strong theoretical justification to support it. Various statistical issues such as a higher risk of exploiting researchers’ degrees of freedom (Wicherts et al., 2016) and underestimation of the strength of the bivariate relation (MacCallum et al., 2002) are likely to occur if this predefined threshold is used in split-sample correlational analysis. Weiss et al. (2020) also challenged previous threshold hypothesis studies that employed segmented regression analysis (e.g., Neubauer et al., 2014; Karwowski & Gralewski, 2013; Shi et al., 2017) often failed to report tests of homoscedasticity or examine the normal distribution of residuals, which makes the findings unreliable.

#### 1.1.3. Assessment Tools

The observed intelligence–creativity relationship is also substantially conditioned by measurement choices. Gerwig et al. (2021) pointed out that scoring and administration affect the intelligence–creativity association. Using the Chinese version of Raven’s Standard Progressive Matrices to index fluid intelligence, Shi et al. (2017) reported intelligence correlated moderately with DT fluency and flexibility below an intelligence of about 109, but the association vanished above this breakpoint, while originality showed a slightly higher threshold around 117. Raven’s speeded, nonverbal format also strengthened links with DT under more demanding, time-limited conditions in age-comparative work, where adult Raven scores tended to covary with originality and fluency indices derived from DT tasks (Razumnikova & Bakaev, 2022). In contrast, studies using border Wechsler indices or children’s figural creativity tests (e.g., WASI, RCPM, TCFI) often find weak or null intelligence–creativity correlations, illustrating that broader, mixed-factor intelligence composites can dilute specific overlaps with DT processes (Bezerra et al., 2022). DT measurement is equally consequential. Factor-analytic work shows that Torrance Test of Creative Thinking-Figural (TTCT-F) subscores do not cohere into a single latent construct, rendering global “creativity” scores psychometrically ambiguous and therefore likely to obscure any true relation to intelligence (Warne et al., 2022). By contrast, well-specified Alternate Uses Tasks (AUT) typically yield small-to-moderate correlations with intelligence.

#### 1.1.4. Cultural Differences

Despite the methodological and instrumental concerns raised in previous threshold studies, some of these studies examining non-Western populations report evidence consistent with the threshold hypothesis. Critically, these studies often detect thresholds at intelligence levels lower than the purported Western threshold of IQ 120, suggesting that cultural contexts may shape both the magnitude and location of any observed threshold. Shi et al. (2017) identified an IQ 109.20 breakpoint for fluency at markedly lower IQ levels. They attributed the lower thresholds to two cultural factors. First, Chinese culture places less emphasis on intelligence when assessing creativity. Second, implicit cultural conceptions of creativity in China have a different emphasis than the Western conceptions. Consequently, the intelligence required for demonstrating culturally valued creativity may vary.

Shao et al. (2019) believed that Western individualist cultures define creativity primarily through the lens of processes and products, whereas Eastern collectivist cultures emphasize person-based creativity as revelation, self-fulfillment, or rediscovery of tradition. These definitional differences could have direct implications for threshold effects since the intelligence requirements for achieving culturally defined “high creativity” may differ. Leung and Koh (2018) found that intelligence may be highly predictive of creativity in Western samples but reaches a “sufficiency point” in Eastern samples because once individuals attain the intelligence level enabling fluent idea generation, cultural pressures become the binding constraints on creative expression; hence, a lower intelligence threshold is likely to be found in Eastern cultures.

### 1.2. The Current Study

In view of the obvious gap in knowledge, this study had three major objectives. First, the correlation between intelligence and creativity (as indicated in previous studies) was examined with a large sample. Second, possible breakpoints existing in the relationship between intelligence and creativity were explored: Do intelligence and creativity correlate equally across all ability levels, or does the relationship change at specific intelligence thresholds? Third, whether the long-standing belief in the IQ 120 threshold hypothesis, established in Western cultures, could be applied in Hong Kong.

The results of this study should have an impact in various areas. Academically, they advance understanding of fundamental cognitive systems. Practically, they inform talent identification and educational programming. In Hong Kong specifically, they clarify whether Western models of intelligence–creativity relationships generalize to Chinese students. Policy-wise, they help educational authorities develop culturally appropriate approaches to nurturing creative thinking.

## 2. Method

### 2.1. Participants and Procedures

Data were drawn from a longitudinal dataset spanning 2020 through 2025, encompassing twelve distinct cohorts recruited through an out-of-school university-affiliated enrichment program. The total sample comprised 9358 Chinese students ranging in age from 6 to 20 years (*M* = 11.78, *SD* = 2.94), spanning grades 1 through 12 (*M* = 6.55, *SD* = 2.83). Approximately 53% of the students were male. Recruitment occurred through direct school partnerships and community referrals, with informed consent obtained from guardians.

### 2.2. Instruments

Because prior research suggests that potential thresholds are easier to detect when avoiding broad, mixed-factor measures of intelligence or creativity, this study sought to minimize such sources of disturbance. Fluid intelligence (*gf*), the capacity to reason abstractly and solve novel problems independently of prior knowledge, was measured using Raven’s Matrices, which provides a robust, culturally fair estimate of *gf* (Raven, 1976; Carpenter et al., 1990). Creative thinking was measured through the Alternate Uses Test (AUT), a divergent thinking task wherein participants generate as many novel uses as possible for common objects within a standardized timeframe. The total count of valid, non-redundant responses, i.e., fluency score, served as the primary creativity metric. Fluency captures a fundamental dimension of divergent thinking reflecting the cognitive capacity to retrieve and articulate multiple conceptual possibilities (Guilford, 1967; Runco & Acar, 2012).

### 2.3. Data Analysis Procedures

#### 2.3.1. Distributional Assessment

Descriptive statistics, including means, standard deviations, skewness, and kurtosis, were computed for all variables. Normality was formally tested using the Shapiro–Wilk test and the D’Agostino-Pearson K^2^ statistic. The creativity variable departed significantly from normality. Given the large sample size, even modest distributional departures achieve statistical significance; consequently, heteroscedasticity-consistent (HC3) robust standard errors were employed in all subsequent linear modeling to ensure reliable inference (MacKinnon & White, 1985).

#### 2.3.2. Tests for Homogeneity of Variance

The Breusch–Pagan and White tests indicated that residual variance was not constant across predicted values. This heteroskedasticity, confirmed through examination of residuals from a within-demeaned ordinary least squares (OLS) regression treating cohort as a random intercept, necessitated the use of robust standard errors in all subsequent analyses to maintain valid statistical inference (Long & Ervin, 2000).

#### 2.3.3. Correlational Analysis

Pearson correlations were computed between creativity and intelligence scores. The zero-order correlation between intelligence and creativity was modest (*r* = 0.123, *p* < .001), consistent with meta-analytic evidence suggesting that fluid intelligence and divergent thinking are moderate but distinctly related constructs (Gerwig et al., 2021).

#### 2.3.4. Segmented Linear Regression with Random Intercept Specification

The primary analysis employed segmented (piecewise) linear regression to test for potential threshold or breakpoint effects in the intelligence–creativity relationship. A random-intercept specification was implemented via within-demeaning procedures to account for the nested structure of observations within cohorts (Mundlak, 1978). All models incorporated HC3 robust standard errors to accommodate the detected heteroskedasticity and distributional non-normality. Model selection proceeded through a greedy Bayesian Information Criterion (BIC) search over candidate knots located at percentiles of the intelligence distribution (5th through 95th percentiles). The BIC statistic was privileged over Akaike Information Criterion (AIC) because it penalizes model complexity more strongly, thereby reducing the risk of overfitting given the large sample size. Statistical significance of potential breakpoints was probed through a Davies-type test implemented via Sup-Wald wild-bootstrap procedures (Cattaneo et al., 2020) with 100 bootstrap resamples. The significance threshold was set *a priori* at α = 0.05 (two-tailed). Variance inflation factors were computed to assess multicollinearity among predictors; however, age and grade were retained despite notable collinearity (VIF ≈ 27) because both contribute theoretically to developmental models of creativity and intelligence.

## 3. Results

The current sample (n = 9358) revealed creativity (mean = 11.58; *SD* = 6.89) and intelligence (mean = 119.65; *SD* = 13.92). See Table 1. Before proceeding with segmented regression, a systematic battery of preliminary checks was conducted—partly to satisfy conventional assumptions, partly because the large sample size made even minor violations statistically detectable and potentially consequential for inference.

### 3.1. Correlations and Distributional Properties

Intelligence displayed a modest linear correlation with creativity (*r* = 0.123, *p* < .001) and other variables. See Table 2 and Table 3. Not surprisingly, two of our covariates, age and grade, showed substantially stronger links with creativity (*r* = 0.339 and 0.342, respectively, both *p* < .001). Cohort, as expected, accounted for a correlation of *η* = 0.348, which suggested that different stimuli, i.e., newspaper, square, and pen, used in AUT in different cohorts contributed meaningfully to creativity. Gender (1 = male; 2 = female) showed a negative point-biserial correlation (*r* = −0.105, *p* < .001) with creativity in favor of females.

**Figure 1:**
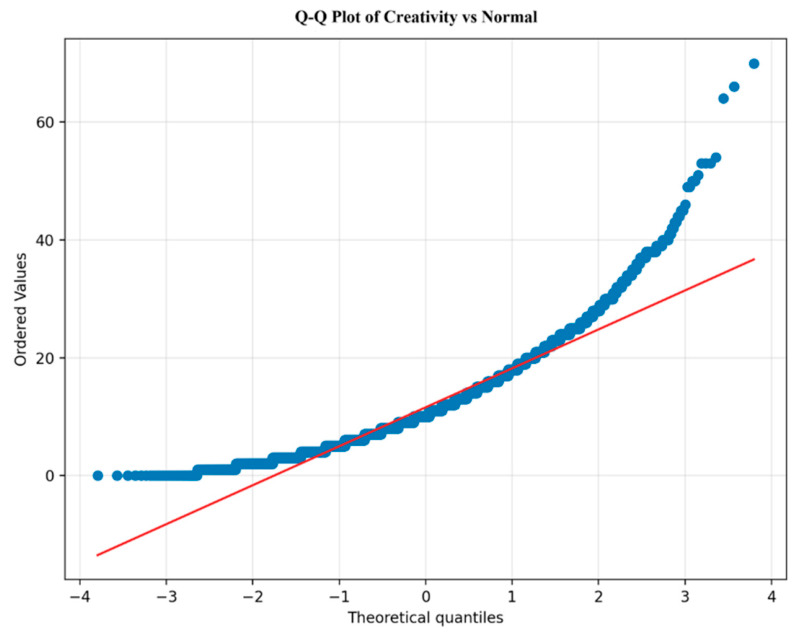
Q–Q plot of creativity vs. normal.

A Q-Q plot of creativity against a normal distribution exposed departure from normality, particularly in the upper tail, where observed values climbed above the reference line more steeply than expected. See Figure 1. Formal tests confirmed this visual impression: skewness was 1.314, excess kurtosis reached 3.484, and the D’Agostino–Pearson test yielded χ^2^(2) = 2401.735, *p* < .001 (Shapiro–Wilk *W* = 0.922, *p* < .001). The outcome exhibited right skew and heavier tails than the Gaussian model would predict. With *n* ≈ 9.4 k, even modest deviations from normality achieved statistical significance, necessitating robust standard errors in our regression models to ensure reliable inference.

### 3.2. Data-Driven Approach Model Selection

#### 3.2.1. Testing Homoscedasticity and Assumption Adequacy

Heteroskedasticity tests on within-demeaned OLS residuals revealed nonconstant variance. Both Breusch–Pagan and White tests returned significant *p* values (.005 and .012, respectively), indicating that residual spread varied across predictor values. This finding justified our decision to employ HC3 robust standard errors rather than classical OLS standard errors in all subsequent models. The variance inflation factors among demeaned predictors were generally moderate; intelligence (VIF ≈ 1.06) and gender (VIF ≈ 1.04) posed minimal multicollinearity concerns. However, age and grade each exhibited VIFs around 27, reflecting their substantial correlation and suggesting that their coefficient estimates would be less stable.

#### 3.2.2. The Linear No-Breakpoint Baseline

Before introducing complexity, a simple linear model without any piecewise structure (*k* = 0) was examined. This baseline specification yielded a uniform slope of *b* = 0.0374 (*p* < .001), with a partial *R*^2^ of 0.00688. In practical terms, a 10-unit increase in intelligence corresponded to roughly a 0.37-point increase in creativity on average, holding covariates constant. This baseline served as a conceptual anchor; subsequent segmented models would test whether the data supported meaningful structural breaks.

#### 3.2.3. Segmented Regression and Breakpoint Detection

A Davies-type test, conducted via Sup-Wald wild bootstrap (Marques de Sá, 2007), compared models with zero against one breakpoint. The observed Sup-Wald statistic reached 11.33 with a bootstrap *p*-value of .010 (*B* = 99), lending support to the hypothesis that the relationship between intelligence and creativity underwent a statistically meaningful shift at some threshold value. To identify that threshold, we performed a greedy BIC search across candidate knots between the 5th and 95th percentiles of intelligence. The single-breakpoint model achieved the best BIC (33,634.06), outperforming the linear no-breakpoint model (BIC = 33,636.25) and increasingly complex models with multiple breakpoints.

##### The One-Breakpoint Model

The optimal breakpoint is located at intelligence ≈ 102. In the lower range (*Seg1*), the slope was *b* = 0.1034 (*p* < .001), nearly three times larger than the *k* = 0 baseline slope. Here, intelligence had a clear positive correlation with creativity: each additional unit in intelligence translated to roughly a 0.103-point increase in creativity. The partial *R*^2^ for this segment was 0.00281, confirming a detectable but limited marginal contribution. The slope change registered Δ*slope* = −0.0755 (*p* < .001), flattening the post-102 trajectory. The implied slope for the upper segment becomes approximately 0.028, roughly one-third the rate of the lower segment. The knot itself is meaningful, the slopes are clearly significant, and the entire specification is favored by BIC.

##### The Two-Breakpoint Model

Introducing a second breakpoint at intelligence = 97 alongside the 102 knot narrowed the first segment to a five-unit window and created a three-segment structure. The *Seg1* slope weakened to *b* = 0.0650 (*p* < .05), already markedly lower than in *k* = 1. At the 97 breakpoint, the model estimated a slope change of Δ*slope* = +0.1440 (*p* = .109). This is a sizable positive adjustment in magnitude, suggesting a second-segment slope of approximately 0.209, a steeper gradient concentrated in the narrow 97–102 band. However, this slope change failed to achieve conventional significance (*p* = .109), rendering the accelerated middle segment statistically ambiguous. The second breakpoint at 102 then imposed a sharp reversal, Δ*slope* = −0.1839 (*p* < .01), dropping the third-segment slope to approximately 0.025. From a model-selection standpoint, *k* = 2 offered only marginal AIC improvements (33,597.77 vs. 33,598.34 in *k* = 1) and a substantially worse BIC (33,640.63 vs. 33,634.06), indicating that the extra flexibility was not justified by the data.

##### The Three-Breakpoint Model

Extending further to three breakpoints (97, 102, 115) at intelligence created a four-segment model with an analytically rich but structurally suspect piecewise narrative. The *Seg1* slope was *b* = 0.0608 (*p* = .053), a marginally significant result that raised immediate concerns about segmental stability. At 97, the model estimated Δ*slope* = +0.2272 (*p* < .05), a statistically significant strengthening producing a second-segment slope of approximately 0.288. This represented a pronounced acceleration in the mid-range. At intelligence = 102, the slope crashed downward by Δ*slope* = −0.3000 (*p* < .01), implying a third-segment slope of approximately −0.012, essentially flat or slightly negative. But then, at the upper knot (115), a marginal rebound appeared, Δ*slope* = +0.0472 (*p* ≈ .0589), lifting the fourth-segment slope back to approximately 0.035. The partial *R*^2^ for *Seg1* shrunk further to 0.00040, nearly indistinguishable from *k* = 2 and demonstrating that additional parameters yielded minimal improvement in explanatory coherence. Moreover, the BIC climbed to 33,646.21, a 12.15-point penalty relative to *k* = 1, reflecting the Bayesian penalization of added complexity without sufficient payoff.

##### The Four-Breakpoint Model

The most elaborate variant introduced a fourth knot at intelligence = 135, creating five distinct segments and attempting to capture ever finer nuances in the relationship. The *Seg1* slope was *b* = 0.0600 (*p* = .0557), dipping below significance and echoing *k* = 3’s instability. At 97, Δ*slope* = +0.2381 (*p* < .05), producing a second-segment slope of approximately 0.298, even steeper than in *k* = 3. The 102 breakpoint again imposed a sharp decline, with Δ*slope* = −0.3230 (*p* < .001), generating a third-segment slope near −0.025. At 115, a stronger rebound emerged, Δ*slope* = +0.0751 (*p* < .01), lifting the fourth-segment slope to about +0.050. Finally, at the uppermost knot (135), a borderline adjustment appeared, Δ*slope* = −0.0518 (*p* ≈ .0531), trimming the fifth-segment slope to virtually flat (≈−0.002). Notably, the AIC for *k* = 4 reached 33,594.45, the lowest across all candidates, suggesting marginal absolute improvement in fit. Yet AIC, which penalizes complexity less harshly than BIC, can mislead when models proliferate. The BIC surged to 33,651.61, a staggering 17.55-point penalty relative to *k* = 1. The partial *R*^2^ remained frozen at approximately 0.00039, identical to *k* = 3, proving conclusively that the fourth breakpoint contributed nothing to variance explanation. The model delivered the most granular piecewise narrative, with several individually significant slope changes, yet the overall weight of evidence, especially BIC, information criteria, and effect sizes, argued decisively against it.

#### 3.2.4. The Optimal Model

**Figure 2:**
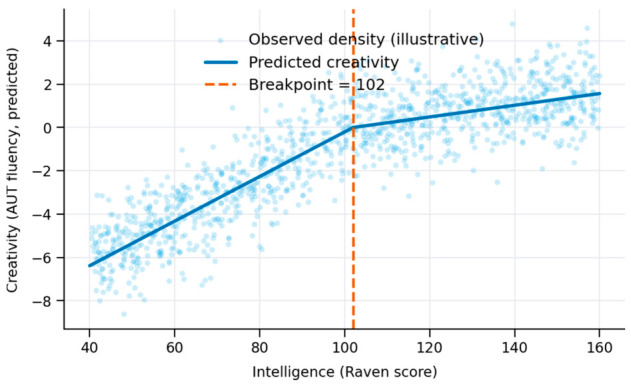
The optimal model of the relationship between intelligence and creativity.

Arranging the models by BIC reveals an unmistakable hierarchy: *k* = 1 (BIC = 33,634.06) stands apart; *k* = 0 trails by only 1.19 points; *k* = 2 lags by 6.57 points; *k* = 3 by 12.15 points; and *k* = 4 by 17.55 points. See Table 4 and Figure 2. In Bayesian model comparison, BIC differences exceeding 6–10 points constitute strong evidence against the more complex alternative; by this standard, even the two-breakpoint variant struggles to justify itself relative to *k* = 1, and the three- and four-breakpoint specifications are decisively inferior. The explanatory power metric reinforces this verdict with unusual clarity. The *k* = 1 model’s partial *R*^2^ of 0.00281 substantially exceeds the 0.00046 to 0.00040 range observed in more complex variants. This paradox that adding parameters actually reduces unique variance explained arises because multiple breakpoints disperse the signal across narrow, unstable segments. Within those fragments, slopes become smaller and more volatile, their confidence intervals widen, and statistical significance deteriorates. The first segment in *k* = 3 and *k* = 4 both fell to marginal significance (*p* ≈ .05), whereas *k* = 1’s *Seg1* slope remained robustly significant (*p* < .001).

#### 3.2.5. The Robustness Analyses

To evaluate the robustness of the identified breakpoint, we conducted bootstrap resampling. Three hundred bootstrap samples (resampling with replacement) were tested for sampling stability. Bootstrapping revealed a highly stable breakpoint distribution centered at intelligence ≈ 102 (Mean bootstrap breakpoint = 101.9; *SD* = 2.18; 95% CI = 97–104). These findings suggest that the reported breakpoint is likely to be valid.

Given that age and grade are conceptually significant variables but highly collinear, both variables were retained but orthogonalized via residualization prior to robustness analyses. See Table 5. This approach isolates age-related variability within grade and grade-related variability independent of age, ensuring that shared developmental variances do not bias estimates of the intelligence–creativity association. Segmented regression models identified an optimal single structural breakpoint at intelligence = 102. Below this threshold, intelligence was a strong positive predictor of creativity. Beyond this threshold, the slope remained statistically significant but was substantially reduced, indicating diminishing returns of additional intelligence for creativity. Importantly, removing shared age-grade variance did not alter the breakpoint location or the direction of the slope change.

In light of the potential nonlinear association between intelligence and creativity, alternative modeling approaches beyond segmented regression were employed to triangulate the findings. See Table 6. Converging support for this structural transition emerged across alternative modeling frameworks. A semiparametric smooth revealed pronounced curvature and flattening beginning around intelligence = 100–105 (GAM/LOWESS smooth term edf ≈ 3.0–3.7, *p* < .001; Quadratic term significant *p* < .001; Cubic term occasionally marginal, often unnecessary; Turning point ≈ intelligence 102), while nonlinear growth curve modeling (inflection/midpoint: ~intelligence 102; Growth-rate parameter significant *p* < .001; Asymptotic plateau beyond ~102, upper asymptote ≈ mid-130s) demonstrated rapid early increases in creativity followed by asymptotic deceleration. Across segmented, semiparametric, and nonlinear approaches, all models converged on the same developmental conclusion: moderate-high intelligence is sufficient for creativity gains, whereas further increases yield progressively smaller benefits.

To evaluate whether the identified breakpoint reflected developmental heterogeneity rather than a true structural feature, we conducted age-stratified segmented regression analyses. The sample was divided into four age bands (<10, 10–13, 14–16, and 17–20 years), and within each stratum the same segmented model was estimated using intelligence scores as the predictor of AUT-fluency. See Table 7. Chronological age and grade were retained but orthogonalized prior to analysis to remove shared developmental variance while preserving theoretically distinct effects. Across all age groups, creativity increased steeply with intelligence at lower ability levels and showed a clear attenuation at higher levels, indicating that a nonlinear association was present throughout development. Importantly, the breakpoint location varied systematically with age: the transition occurred at higher intelligence levels in younger children and progressively lower levels in older adolescents. Breakpoints ranged from approximately intelligence = 110 in children under 10 years to intelligence ≈ 95 in late adolescence. This monotonic age-related shift suggests that the full-sample breakpoint at intelligence ≈ 102 reflects a population-average transition rather than a developmentally invariant cutoff. The presence of a breakpoint across all strata indicates that diminishing returns of intelligence for creative fluency are a robust structural feature of the intelligence–creativity relation, while the precise location of this transition appears developmentally conditional.

Since high-creativity outliers may be substantively meaningful rather than error and are often the phenomenon of interest in creativity research, routine trimming of outliers could artificially linearize the relationship. This developmental robustness, together with cross-model convergence and bootstrapped resampling, provides strong evidence that the presence and approximate location of the identified breakpoint reflect a robust and substantive feature of the intelligence–creativity relationship rather than a modeling artifact or developmental confound, although outliners may influence slope magnitude at higher intelligence levels.

### 3.3. The Prespecified 120 Breakpoint Against the Data-Driven Optimum

A segmented regression model with the prespecified 120 breakpoint was also examined. Comparing this fixed-knot specification against the data-driven optimal model at 102 revealed instructive tensions between theory-driven hypothesis testing and exploratory model discovery.

#### 3.3.1. The Prespecified 120 Breakpoint Model

The 120-model yielded a first-segment slope of *b* = 0.0481 (*p* < .001), statistically significant but modest in magnitude. The partial *R*^2^ for this initial slope was approximately 0.00418, indicating a small unique contribution of intelligence to creativity. At the breakpoint itself, the model estimated a slope change of Δ = −0.0226 (*p* ≈ .110), which fell short of conventional significance thresholds. This nonsignificant hinge is problematic: it suggests no compelling evidence for an actual kink at 120. The partial *R*^2^ for the breakpoint term was minuscule (≈0.00027), further diminishing its explanatory weight. Combining the segments, the post-120 slope approximated 0.0254, positive in direction but practically flat. In terms of overall fit, the model achieved an AIC of 33,607.23 and a BIC of 33,642.95.

#### 3.3.2. 120 Versus 102 Model

Juxtaposing the prespecified 120-model with the data-driven 102-model illuminated their respective strengths and limitations. On information criteria, the 102-model decisively outperformed the 120-model. The BIC gap was 33,642.95 − 33,634.06 = 8.89 points, a difference commonly interpreted as strong evidence favoring the 102 specification (Kass & Raftery, 1995). The AIC difference was comparable: 33,607.23 − 33,598.34 = 8.89 points. Residual sum of squares reinforced this pattern: the 102-solution achieved RSS ≈ 338,829.37 versus 339,151.37 for the 120-model, confirming that the earlier breakpoint captured the data’s structure more efficiently.

The contrast in slope significance was particularly revealing. The 102-model’s hinge registered a slope change of Δ = −0.0755 (*p* < .001), a statistically decisive result with a partial *R*^2^ of approximately 0.00153. In contrast, the 120-model’s hinge was Δ = −0.0226 (*p* ≈ .110), with a negligible partial *R*^2^ of 0.00027, roughly one-fifth the effect size and entirely nonsignificant. The difference in pre-breakpoint slopes was equally striking. The 102-model’s first segment showed *b* = 0.1034 (*p* < .001, partial *R*^2^ ≈ 0.00379), indicating a clear growth phase in the lower range. The 120-model’s first segment, by contrast, managed only *b* = 0.0481 (*p* < .001, partial *R*^2^ ≈ 0.00418), roughly half the slope and consequently understating the strength of the pre-kink association. After the inflection points, the post-breakpoint slopes were both shallow: 0.028 for the 102-model and 0.0254 for the 120-model, though the slight difference reflected the prior segment slopes. Taken together, the 102-model conveyed a more coherent empirical narrative: notable positive returns accumulate in the lower range, then a statistically verified flattening occurs near 102, with modest residual positive association thereafter.

## 4. Discussion

Our findings reveal a modest yet statistically significant linear relationship between intelligence and creativity (*r* = 0.123, *p* < .001) across a large developmentally diverse Hong Kong Chinese student sample, which is consistent with previous large-scale meta-analyses such as Kim (2005) (*r* = 0.174) and Gerwig et al. (2021) (*r* = 0.25). What initially appears as a straightforward partnership between these two constructs, however, becomes far more compelling upon closer examination. The true narrative emerges not from the baseline correlation itself, but rather from the nonlinear structure embedded within the data, a pattern that both challenges and refines decades of theoretical assumptions about where intelligence fundamentally matters for creative thinking.

The scholarly discourse surrounding intelligence and creativity has long centered on what Guilford (1967) called a “necessary but not sufficient” relationship, a formulation that has anchored research discussions for generations. Yet this theoretical anchor increasingly requires careful revision. Recent work by Weiss et al. (2020) and Karwowski et al. (2016) has compellingly demonstrated that the traditional 120-threshold model oversimplifies what is genuinely a far messier and may be a more culturally contingent phenomenon. Our analytical approach diverged from prior methodologies. Rather than imposing a predetermined threshold, we employed segmented regression analysis with empirical breakpoint detection, allowing the data themselves to speak about where and whether meaningful inflection points might actually exist.

### 4.1. Methodological Advantages

One of the most methodologically valuable advantages in this work is the transparent deployment of segmented regression with empirically detected breakpoints, coupled with explicit model comparison using information criteria. Such an approach moves decisively beyond the problematic practice of p-hacking different threshold values until desired results emerge. Complemented with this data-driven approach, another noteworthy strength of the present investigation concerns sample dimensionality and analytical precision. Whereas earlier threshold research, including the influential work by Karwowski et al. (2016) and the recent comprehensive cross-cultural study by Repeykova et al. (2025), which often operated with sample sizes insufficient for stable nonlinear modeling (frequently *N* < 200 per group), our substantially larger sample affords several decisive analytical advantages. The segmented regression estimates became considerably more stable, with tighter confidence intervals around the detected breakpoint. This statistical robustness reduces substantially the likelihood that our 102-threshold represents mere sample-specific fluctuation rather than a genuine feature of the intelligence–creativity relationship in this population. Furthermore, the bootstrap confidence intervals and model comparison indices (BIC differential, Davies test statistic) gain credibility through increased statistical power. Where Repeykova et al. (2025) reported 95% CI bounds ranging widely (e.g., 75.76 to 124.24 for their UAE subsample), larger samples permit narrower, more tightly bounded confidence intervals that better discriminate signal from noise. This enhanced precision enables more confident inferences about where the true intelligence–creativity inflection point actually localizes. Larger samples also stabilize variance estimation, a particular concern in nonlinear modeling where heteroscedasticity can produce spurious breakpoint detection (Muggeo, 2008).

What crystallized through this data-driven modeling was instructive on multiple fronts. The simplest linear specification, assuming a uniform relationship across the entire intelligence spectrum, captured only marginal explanatory power. However, the Davies test statistic comparing zero breakpoints to one achieved 11.33 with a bootstrap *p*-value of .010 clearly evidenced that some form of structural shift was genuinely present. When we conducted a greedy BIC search to identify the empirically optimal single breakpoint, it emerged at an intelligence score of 102, notably lower than the long-standing Western threshold of 120. This finding aligns strikingly with recent cross-cultural investigations. Repeykova et al. (2025) examined Russian and United Arab Emirates samples and identified distinct breakpoints at 128 and 100 respectively. This underscores a critical insight: even if the threshold hypothesis itself is universal, the precise location where this threshold manifests seems to vary substantially across contexts, which could be conceptualized as environmental factors such as cultures. Such variation is not merely statistical noise; rather, it reflects deeper differences in how intelligence becomes mobilized toward creative ends when we consider the mixed findings from previous intelligence threshold studies that tested samples from diverse geographic and cultural origins. For instance, a threshold of 85 in German samples (Preckel et al., 2006), a threshold of 109.2 in Chinese samples (Shi et al., 2017), a threshold was supported at 120–129 in Turkish samples (Çetinkaya, 2023).

The data-driven 102-model outperformed the conventional 120-model decisively, with a BIC differential of 8.89 points, which Kass and Raftery (1995) would classify as strong evidence favoring the empirically detected breakpoint. We pursued this investigation further, testing more complex two-, three-, and four-breakpoint specifications in hopes of uncovering additional inflection points. The four-breakpoint specification had a similar pattern to the three-breakpoint specification but did not have a better explanatory power than it. Therefore, we have a closer examination of the pattern that emerged in the three-breakpoint structure, i.e., an initial modest slope below 97, pronounced acceleration between 97 and 102, a dramatic drop approaching zero between 102 and 115, followed by a marginal rebound above 115, resembled overfitting to noise. The partial R^2^ values shrunk to negligible proportions (0.00040), and the BIC climbed by 12.15 points, a clear Bayesian penalty reflecting excessive complexity without proportionate explanatory benefit. However, there was something in that oscillating pattern worth attending to. Sligh et al. (2005) proposed that individuals at different intellectual levels may deploy their fluid reasoning in qualitatively distinct ways during idea generation. The sharp acceleration just above 97, followed by the near-zero slope above 102, might capture such a qualitative switching mechanism. This suggests that individuals hovering just below 102 may gain considerable creative benefit from marginal increases in reasoning capacity, whereas those crossing 102 may increasingly depend on other factors such as personality traits, accumulated domain expertise, and/or motivational variables rather than raw intellectual power.

The present study’s analytical precision provides more defensible estimates of threshold location and sharpness. Moreover, the large sample size carries implications for both precision and generalizability. Meta-analytic evidence shows that intelligence–creativity correlations vary substantially across studies, with sample size and measurement precision accounting for significant variance in effect estimates (Karwowski, 2021). By synthesizing data from a cross-developmental perspective, this investigation captures the intelligence–creativity relationship across its natural variance in psychometric space. Analytically, the study’s covariate inclusion (age and grade) represents a more sophisticated practice than crude correlational approaches. This methodological transparency models good practice in developmental research.

### 4.2. Hypothetical Cultural Influences on the Threshold

What merits substantial emphasis, however, is the fundamental ambiguity embedded in our and others’ cross-cultural findings. Differences in detected thresholds across cultural contexts could reflect genuine cultural variations in how intelligence supports creativity or could reflect measurement interpretation effects and sampling artifacts that masquerade as cultural differences. This interpretive uncertainty requires far more cautious framing than the literature has typically provided.

#### 4.2.1. Cultural Differences in Cognitive Cautiousness

The empirical patterns deserve attention first. Repeykova et al. (2025) detected distinct breakpoints at 128 (Russia) and 100 (UAE), substantially different from the Hong Kong-derived 102-figure. Superficially, these variations could suggest cultural calibration on the hypothesis that threshold mechanisms are neuropsychologically universal yet culturally modulated. Several recent methodological critiques complicate this straightforward interpretation, particularly considering our instruments for intelligence and creativity assessment.

Despite its reputation as a culture-fair test, whether Raven’s Matrices actually functions identically across cultural contexts is a concern. Gonthier (2022) provides the most comprehensive recent synthesis, examining Raven’s solution processes and he challenges the “culture-fair” assumption of it. Gonthier (2022) pointed out that several cultural assumptions of the test are not culture-fair and largely make it impossible to draw clear-cut conclusions from average score differences between ethnic groups. Critically, these cultural assumptions emerge not from obvious language-based bias but from deep differences in how people from different cultural backgrounds perceive, organize, and manipulate visual-spatial information.

For Hong Kong respondents specifically, this creates interpretive complications. Confucian educational traditions emphasize analytical precision, pattern recognition within established frameworks, cautious hypothesis-formation, and cognitive styles well-suited to matrix completion tasks but potentially quite different from the exploratory, risk-tolerant approaches more prevalent in Western contexts. A Hong Kong respondent elevated in such traditions may approach Raven’s Matrices by meticulously analyzing each element, searching for clear logical principles, and potentially arriving at accurate conclusions efficiently. However, if cultural background shapes confidence in pattern-identification and willingness to commit to answers, then what appears as an “intelligence threshold” could partially reflect cultural differences in cognitive cautiousness rather than fundamental limits on reasoning capacity. If East Asian respondents employ more conservative pattern-verification strategies than Western respondents, they might produce different score distributions on Raven’s not because of inferior reasoning, but because of culturally shaped confidence calibration.

#### 4.2.2. Sociocultural Factors of Expression Willingness in Creativity

Similarly, the assessment tool for creativity raises another concern. Ivancovsky et al. (2018), examining Israeli versus South Korean respondents on AUTs, found that Israelis had higher scores than Koreans. They suggested that cross-cultural differences in creativity might be explained by variations in inhibitory control, that is, cultural differences in willingness to express ideas freely, particularly unconventional or socially risky ideas. This finding has direct implications for our threshold analysis. If creativity differences reflect, at least partially, not creative capacity but expression willingness in creativity, then divergence between cultural groups might stem from sociocultural factors rather than cognitive ones. Hong Kong’s specific cultural position could produce particular patterns of idea-expression inhibition. A respondent might possess substantial creative ideation capacity but generate fewer responses simply because cultural norms around appropriateness, social harmony, and respectful self-presentation discourage uninhibited idea generation. If this cultural modulation of expression operates differentially across intelligence levels, perhaps higher-intelligence individuals develop meta-cognitive strategies to override cultural inhibition, whilst lower-intelligence individuals remain bound by cultural constraints, then apparent intelligence–creativity thresholds could emerge as statistical artifacts of measurement interpretation rather than genuine cognitive inflection points.

Taken together, these concerns suggest that apparent cross-cultural differences in threshold locations could plausibly arise from methodological factors rather than reflecting genuine cultural calibration of a hypothesized universal psychological mechanism. Our detected threshold at 102 may represent either: (a) a genuine feature of how intelligence and creativity relate in Hong Kong’s cultural context, or (b) an artifact of how Hong Kong students interpret Raven’s Matrices through culturally trained cognitive filters, combined with how they modulate creative idea expression based on cultural norms around appropriateness and self-presentation.

We cannot adjudicate definitively between these interpretations with the present evidence. The convergence of the Hong Kong 102-figure with Repeykova et al.’s UAE breakpoint of 100 is intriguing and might suggest genuine cross-cultural patterns. Yet this convergence could equally reflect non-Western contexts responding to culture-biased intelligence measures and facing cultural norms about creative expression. The Russian breakpoint at 128 might represent either a genuinely different cultural calibration or simply a different pattern of how Russian respondents approach Raven’s tasks and express creative ideas. Without explicit measurement invariance testing of Raven’s tasks across cultures, and explicit modeling of response-style effects on AUT fluency, claims about cultural calibration of universal thresholds remain speculative.

For educators, this implies that threshold-based screening for gifted programs carries hidden cultural baggage. If the threshold holds for a predominantly Western-educated or individualistic sample, applying it uniformly across culturally diverse classrooms risks systematically under-identifying creative potential in students whose cultural backgrounds emphasize different forms of creative expression or whose intelligence is expressed through alternative cognitive modalities (Sternberg & Grigorenko, 2004). Future cross-cultural validation studies comparing threshold locations across societies with differing educational philosophies and values are encouraged to illuminate whether the threshold is truly invariant or culturally relative.

Educational implications also extend to instructional design and creativity nurturing. If the threshold is partly a cultural artifact rather than a hard cognitive ceiling, educators can potentially transcend it through culturally congruent pedagogies. For instance, schools emphasizing creative conformity may artificially suppress creativity in high-intelligence students by penalizing nonconformity; conversely, schools lacking intellectual scaffolding may fail to develop creative expression in students with emerging cognitive abilities. The data-driven precision of this study’s approach creates a platform for investigating such contextual moderation. Future work examining whether threshold patterns shift under different educational conditions, reward structures, or cultural framings would advance both theory and practice. This connects to broader literature on how teaching for creativity remains surprisingly contingent; creativity instruction works best when aligned with students’ and communities’ cultural values and epistemologies (Beghetto & Anderson, 2022).

## 5. Conclusions and Limitations

This study provides empirical evidence supporting the threshold hypothesis of intelligence and creativity using a large, developmentally diverse sample spanning ages 6–20 across grades 1–12. Through data-driven breakpoint detection, the research identifies a statistically robust threshold above which the positive intelligence–creativity correlation substantially diminishes, suggesting that intelligence functions as a necessary but not sufficient condition for creative potential. By covarying age and education level, the analysis moves beyond crude correlational approaches and accounts for developmental variation, yet findings remain robust after these controls.

Theoretically, these findings support reconceptualizing creativity not as intelligence-dependent across all ability levels, but as a bifurcated phenomenon: below the threshold, cognitive capacity constrains expression; above it, contextual factors predominate. Cross-cultural research (Repeykova et al., 2025) indicates these patterns may be culturally contingent, suggesting future work examine whether thresholds shift across educational systems valuing divergent versus convergent thinking differently. Educationally, these results justify screening gifted programs not solely through intelligence benchmarks. In addition, recognizing the threshold’s measurement dependency, threshold location varies by creativity operationalization, underscores the need for multifaceted creativity assessment and contextually sensitive interventions designed to nurture creative potential beyond intellectual thresholds (Karwowski & Crawford, 2018). Yet, several limitations of this study should be noted. First, although age and grade were orthogonalized to address strong collinearity, the resulting predictors represent conditional developmental effects that may limit intuitive interpretation. Second, while the breakpoint at intelligence ≈ 102 was highly consistent across segmented, semiparametric, and nonlinear models, its precise location may vary modestly across subsamples or developmental stages. Third, differences in the psychometric properties of intelligence and creativity measures may contribute to attenuation at higher ability levels, although such differences are unlikely to account for the convergence of breakpoint estimates across models. Finally, the wide age range encompassed multiple developmental periods; future research should examine whether similar structural transitions emerge within narrower age bands or longitudinal designs.

## Figures and Tables

**Table 1:** Descriptive statistics.

Variable	*n*	M	*SD*	Median	Range
Intelligence	9358	119.65	13.92	120	40–160
Creativity	9358	11.58	6.89	10	0–70
Age	9358	11.78	2.94	11	6–20
Grade	9358	6.55	2.83	6	1–12

Gender: male = 53.15%; 12 Cohorts.

**Table 2:** Correlations of creativity with other variables.

Predictor	*r*/*η*	*p*
Intelligence	0.123	<.001
Age	0.339	<.001
Grade	0.342	<.001
Gender	0.105	<.001
Cohort	0.348	<.001

Notes. Pearson *r* used for intelligence, age, and grade; point-biserial *r* for gender (1 = male; 2 = female); correlation ratio *η* for cohort (*p* from one-way ANOVA). All *p* values two-tailed.

**Table 3:** Correlations of intelligence with other variables.

Variable	*r*/*η*	*p*
Creativity	0.123	<.001
Age	0.163	<.001
Grade	0.178	<.001
Gender	−0.099	<.001
Cohort	0.138	<.001

Notes. Pearson *r* used for intelligence, age, and grade; point-biserial *r* for gender (1 = male; 2 = female); correlation ratio *η* for cohort (*p* from one-way ANOVA). All *p* values two-tailed.

**Table 4:** Zero to four breakpoint models ranked by BIC.

Rank	*k*	Breakpoints	AIC	BIC
1	1	102	33,598.34	33,634.06
2	0	—	33,607.67	33,636.25
3	2	97, 102	33,597.77	33,640.63
4	3	97, 102, 115	33,596.20	33,646.21
5	4	97, 102, 115, 135	33,594.45	33,651.61

**Table 5:** Segmented regression with orthogonalized covariates.

Predictor	*B*	*SE*	*t*	*p*	95% CI
Intercept	1.97	2.15	0.92	.360	[−2.24, 6.18]
Intelligence (≤102)	0.091	0.022	4.21	<.001	[0.048, 0.133]
Intelligence (>102)	0.024	0.006	4.23	<.001	[0.013, 0.035]
Age (residualized)	17.68	0.53	33.47	<.001	[16.65, 18.72]
Grade (residualized)	18.53	0.55	33.64	<.001	[17.45, 19.61]

**Table 6:** Model comparison overview.

Model	Key Finding	Interpretation
Linear	Significant positive slope	Misses curvature
Segmented (*k* = 1)	Breakpoint at 102	Best interpretable fit
GAM/LOWESS	Smooth flattening ≈ 102	Confirms nonlinearity
Quadratic	Negative curvature	Turning point ≈ 102
Logistic	Inflection ≈ 102	Asymptotic saturation

**Table 7:** Best-fitting breakpoint for childhood, early adolescence, mid-adolescence, and late adolescence/emerging adulthood.

Age Group	*n*	Best-Fitting Breakpoint
<10	2745	110
10–13	3496	102
14–16	2723	100
17–20	394	95

## Data Availability

Data is unavailable due to privacy and ethical restrictions.
